# The Toxicological Testing and Thermal Decomposition of Drive and Transport Belts Made of Thermoplastic Multilayer Polymer Materials

**DOI:** 10.3390/polym12102232

**Published:** 2020-09-28

**Authors:** Piotr Krawiec, Łukasz Warguła, Daniel Małozięć, Piotr Kaczmarzyk, Anna Dziechciarz, Dorota Czarnecka-Komorowska

**Affiliations:** 1 Institute of Machine Design, Faculty of Mechanical Engineering, Poznan University of Technology, Piotrowo 3 Str., 61-138 Poznan, Poland; piotr.krawiec@put.poznan.pl (P.K.); lukasz.wargula@put.poznan.pl (Ł.W.); 2 Laboratory of Combustion Processes and Explosions, Scientific and Research Center for Fire Protection, National Research Institute, 05-420 Józefów, Poland; dmaloziec@cnbop.pl (D.M.); pkaczmarzyk@cnbop.pl (P.K.); adziechciarz@cnbop.pl (A.D.); 3 Institute of Materials Technology, Polymer Processing Division, Faculty of Mechanical Engineering, Poznan University of Technology, Piotrowo 3 Str., 61-138 Poznan, Poland

**Keywords:** flat drive belts, transport belts, polymer composites, thermal decomposition, combustion, danger during fire, fire protection, FT-IR, morphology, thermogravimetry

## Abstract

The article presents the potential impact of flat drive and transport belts on people’s safety during a fire. The analysis distinguished belts made of classically used fabric–rubber composite materials reinforced with cord and currently used multilayer polymer composites. Moreover, the products’ multilayers during the thermal decomposition and combustion can be a source of emissions for unpredictable and toxic substances with different concentrations and compositions. In the evaluation of the compared belts, a testing methodology was used to determine the toxicometric indicators (W_LC50SM_) on the basis of which it was possible to determine the toxicity of thermal decomposition and combustion products in agreement with the standards in force in several countries of the EU and Russia. The analysis was carried out on the basis of the registration of emissions of chemical compounds during the thermal decomposition and combustion of polymer materials at three different temperatures. Moreover, the degradation kinetics of the polymeric belts by using the thermogravimetric (TGA) technique was evaluated. Test results have shown that products of thermal decomposition resulting from the neoprene (NE22), leder leder (LL2), thermoplastic connection (TC), and extra high top cower (XH) belts can be characterized as moderately toxic or toxic. Their toxicity significantly increases with the increasing temperature of thermal decomposition or combustion, especially above 450 °C. The results showed that the belts made of several layers of polyamide can be considered the least toxic in fire conditions. The TGA results showed that NBR/PA/PA/NBR belt made with two layers of polyamide and the acrylonitrile–butadiene rubber has the highest thermal stability in comparison to other belts.

## 1. Introduction

Flat belts are used in machines and devices fulfilling both drive and transport function [1,2,3]. Classic and commonly used draw belts were made in the form of fabric–rubber composites reinforced with a cord [4,5]. Currently, due to the development of construction materials, they are made of various polymers-based composites, e.g., poly(ethylene terephthalate) (PET), polyamide (PA), polyurethane (PU), and polyoxymethylene (POM) [6]. Polymer composite belts may be divided into three groups: elastic light belts reinforced with fabrics, rigid belts with increased strength with thick films as their cores, and durable flexible belts reinforced with cords [7]. Some of the most common materials from which separate layers are made are polyamide (PA), polyester, polyurethane (PU), rubber, polyvinyl chloride (PVC), aramid or carbon fibers, etc. [8,9]. The literature presents the results of tests of the mechanical properties of uniform flat belts [7,9] as well as tests that take into account the influence of machining (perforation) [10]. However, there is little information containing the effects of high temperature and the susceptibility to inflammation of such ties. During the operation of cable gears as a result of damage to machine components or external factors, the belts are exposed to high temperature [11,12]. An example would be seizing and stopping tensioning pulleys or intermediate pulleys, changing the nature of the cooperation between the pulley and the belt from rolling friction to sliding [13]. The causes of ignition of belts may be mechanical damage to machine elements [14,15], gearbox pollution [16], or external factors affecting high temperature [17].

Nowadays, scientists are testing to not only improve the construction properties of machine parts, but also to limit the effects of fire on machines and devices [18,19]. Solorzano et al. [20] carried out testing on materials intended for facades of prefabricated buildings such as the layers of cross-laminated timber (CLT) and the inner core of aluminium composite panels (ACPs), demonstrating that CLT is more fire retarded than the polymeric internal core of ACP façade materials [20]. Other directions of activities in the construction industry are works on the use of polyurethane foam waste for reuse in ecological building materials [21]; this material is one of the basic materials used inside buildings for upholstery furniture. They are characterized by poor fire resistance; therefore, their testing is the topic of many scientific papers [22]. Xi et al. [23] conducted works on self-hardening polyurethane foam from glucose-based non-isocyanate polyurethanes (g-NIPU), showing that these foams require the addition of flame retardants as in polyurethane foams [23,24]. Many scientific papers concern testing aimed at assessing the flammability and smoke suppression of materials with a wide spectrum of applications, e.g., water-dilutable epoxy resins [25,26], plasticized-poly (vinyl chloride) (P-PVC) for cables and insulation [27]. The rescue and mining industry is conducting testing on flame-retardant rigid polyurethane foam used to suppress fires in coal mines in the field of fire resistance and the improvement of production efficiency [28,29].

Czarnecka-Komorowska et al. developed a method of increasing the thermal resistance of polyoxymethylene (POM) [30], which is a typical engineering polymer through physical modification consisting of the addition of polyhedral oligomeric silsesquioxanes (POSSs) nanofillers with various functional groups [30,31,32,33,34] into the POM matrix. They also developed the technology and method of producing POM/amino POSS composites with reduced emissions of formaldehyde and trioxane compounds from polyoxymethylene [34].

Polyhedral oligomeric silsesquioxanes (POSS) are nanostructures with the empirical formula of (RSiO_1.5_)_n_, where R is a hydrogen atom or an organic group (alkyl, alkylene, acrylate, hydroxyl, or epoxide units) [35,36,37]. These chemicals are composed of a silicon and oxygen cage, which is externally completed by organic groups that are covalently bonded with the silicon atoms. The most common value of n is 8, thus generating a very highly symmetric structure (T8), which has a diameter that is usually in the range of 1.5–3 nm [35,36,37]. POSSs are known from 1946, when they were first described by Scott [36], but the rediscovery of these materials is certainly due to the work of Feher, who set up their synthesis with easily reproducible methodologies [37], and Lichtenhan, who understood the infinite potential of POSSs in being able to be mixed with polymers for making hybrid composites [38].

Numerous studies have been conducted to increase fire resistance and the effects of combustion and thermal decomposition of polymer composites [39,40,41,42]. Tang et al. [39] carried out testing on the properties of pure polypropylene (PP) and basalt fiber-reinforced polypropylene (BFRPP) during combustion. Tang et al. [39] showed that adding basalt fibre to pure polypropylene (PP) can reduce the maximum thermal decomposition rate by increasing the temperature at a maximum weight loss rate. Adding basalt fiber to PP could slightly reduce the limiting oxygen index [39]. Barczewski et al. [40] indicated that basalt powder can be used as a filler in order to increase the thermal stability and reduce the flammability of polypropylene composites. Sheng et al. [41] described the method of suppressing fire and smoke during the combustion of polyvinyl alcohol (PVA) through the synergistic effect of ammonium polyphosphate (APP), and transition metal carbide (MXene) composite aerogels were prepared via the freeze-drying method. Tests of such materials are usually carried out employing thermogravimetric analysis (TGA) and pyrolysis combustion flow calorimetry (PCFC). Arrhenius parameters and the associated calorimetric quantities, i.e., heat release rates, temperature to the peak heat release rate, heats of combustion, heat release capacities, and char yields, were also evaluated [41].

Nowadays, fiber-reinforced composites are commonly used materials for tension belts. Such material not only provides a high strength-to-weight ratio but also exhibits unique properties such as high strength, stiffness, attenuation property, flexural strength, and resistance to corrosion, wear, impact, and fire [40,41,42]. And, more particularly, to an aramid fiber (PA) cord for use as a load carrying component in a power transmission belt and having excellent resistance to flexing fatigue, deformation, and fraying [42]. The aramid fiber (PA) is an organic fiber that is strong and flexible and has good dimensional stability in high temperature environments as compared to other organic fibers [42]. An aramid fiber cord for use as a component in a power transmission belt, which aramid fiber cord has a plurality of PA fiber filaments of 300 to 3100 denier adhesively treated and formed into a strand [42]. This wide range of various functions meant that composite materials were used in the electromechanical, construction, aerospace, automotive, biomedical, and marine and many other industries [42,43,44,45,46,47]. As a literature review shows, the composites of these materials are not studied in terms of chemical compound emissions during thermal decomposition and combustion.

The aim of this paper is an assessment of the toxicity of thermal decomposition products of flat belt combustion from the polymer materials used. The results were to the thermogravimetry (TGA) and the toxicometric indicators (W_LC50M_) with the classification of materials according to the thermal decomposition and combustion products in terms of their toxicity.

## 2. Materials and Methods

### 2.1. Materials

**Figure 1:**
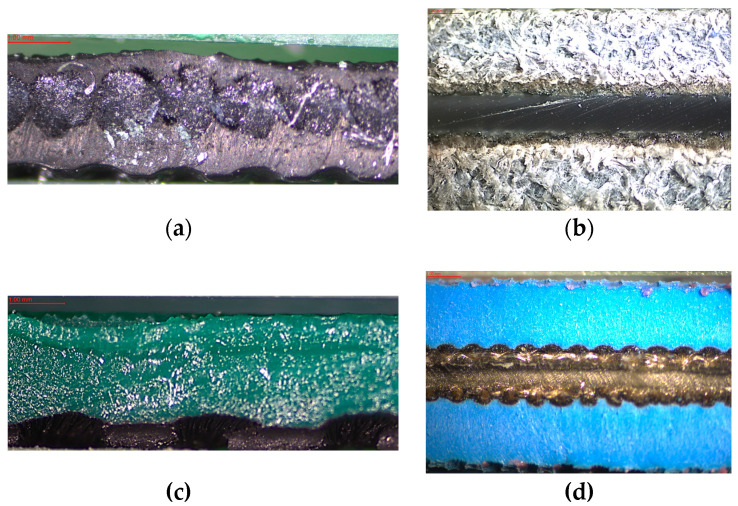
Optical light microscopy micrographs of flat belts: (**a**) NE22, (**b**) LL2, (**c**) TC, and (**d**) XH (magnification 20×).

For toxicological testing, and to determine toxicometric indicators, four types of drive and transport belts were investigated. The belts made of different materials such as classic material named neoprene (abbreviation NE22) and polymer composite materials belts used nowadays such as leder leder (abbreviation LL2), thermoplastic connection (abbreviation TC), and extra high top cower (abbreviation XH) were compared. The NE22 belt was produced by a Brecoflex [45], and LL2 belt was manufactured by a Chiorino) [46]. The conveyor and transmission TC and XH belts were produced by a Nitta (Osaka, Japan) [47]. All selected belts had an average specific density approx. of 1.2 g/cm^3^ and average hardness Shore of about 80 ^o^A. The morphology of belts composites was observed using optical light microscopy (PLM), as shown in Figure 1.

Selected physical properties of multilayers polymer composites belts are listed in Table 1.

The classic belt marked as NE22 was made of a layer of woven polyester and covered with polychloroprene (Figure 1a). The volume ratio of woven polyester to polychloroprene in belt NE22 was 1.0 to 3.0. It is resistant to abrasion, with a high friction coefficient, good resistance to oils, greases, and ozone. It exhibits antistatic properties and high flexibility, and it can be used on pulleys with small diameters. It is used in the temperature range from −20 to 100 °C and a linear speed up to 100 m/s, in high-speed drives that occur in the textile industry (spindle drives), the paper industry, and printing houses, as well as simple drives that do not require high kinematic efficiency.

The LL2 flat belt, which was shown in Figure 1b is made of three alternating layers of leather and polyamide 6 (PA6). The volume ratio of leather to polyamide 6 in belt LL2 is 4.0 to 1.0. These belts are characterized by good resistance to variable loads, and short-term permanent slip, they have good cooperation with the wheels, and they show antistatic properties. These belts can be used for operation in the temperature range from −20 to 100 °C, in mills, chippers, machines, and equipment intended for woodworking.

The TC belt was made of two polyurethane (TPU) layers, lower black (TPU) with a rough structure and upper green (conductive TPU) with a smooth structure (Figure 1c). The black surface is the running side of the belt, while the dorsal side can be used for transport, e.g., in the textile industry. Such tendons are used in drives with high movement speeds, and due to the installation with limited access to the belt, these can be easily joined to obtain a closed belt. The operating temperature range of the TC belt is from −20 to 60 °C, and it can achieve linear speeds up to 40 m/s; thanks to the significant extension, the belt can be placed on wheels without a tensioner. The TC belts are used in printing and textile industries, in drives where it is not possible to introduce preload.

The XH belt was built of four polymers layers (Figure 1d). The upper and lower are (acrylonitrile–butadiene rubber)—NBR of 70%, middle layers are polyamide (PA) film of 15%, and polyamide (PA) fabric of 15%. Special abrasion-resistant synthetic rubber (NBR) is used for the belt surface offering stable coefficient of friction and high abrasion resistance, and high-quality oriented polyamide film is used for the tension member to offer high tensile strength [46]. The belt is characterized by excellent abrasion resistance, high elasticity, high efficiency, long working time, and it is maintenance-free. Moreover, the XH belt demonstrates resistance to oils and humidity. It can be used in temperature range from −20 to 80 °C, in printing houses (folder–gluer) in the production of packaging.

**Figure 2:**
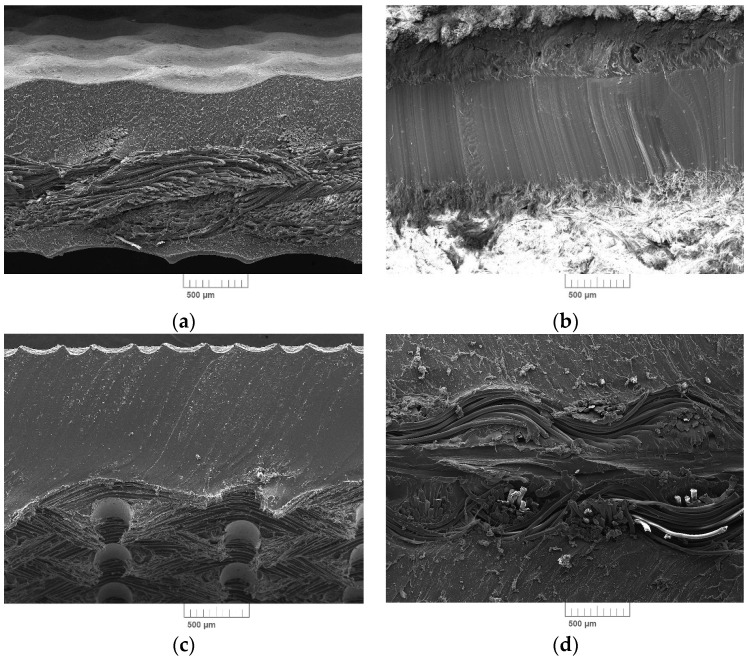
SEM pictures of flat belts: (**a**) NE22, (**b**) LL2, (**c**) TC, and (**d**) XH.

SEM microscopic images of the fracture surfaces of the tested belts are shown in Figure 2.

### 2.2. Methods

#### 2.2.1. FT-IR Spectroscopy

To evaluate the toxicity of combustion and chemical decomposition of the belts, the Fourier-transform infrared spectroscopy (FT-IR) method was used. The measurements were carried out using a Jasco FT/IR 4700 apparatus (Tokyo, Japan). IR spectra were recorded in the spectral range of 4000–500 cm^−1^ with a resolution of 4 cm^−1^ and an average number of scans of 16. Spectroscopic data were treated using the dedicated software Spectra Manager (ver. 2, Jasco, Easton, MD, US).

#### 2.2.2. Density and Shore Hardness

The solid masses of the belts were measured by an electronic balance (AXIS AD50-AD200, AXIS, Gdansk, Poland). The density was measured based on PN-EN ISO 1183-1:2005 standards [48]. Ethyl alcohol (as an immersion liquid) was used, and measurements were made for five samples from each series. The hardness of belts was also measured using a Shore A hardness tester (Sauter HBD 100-0 GmbH (Balingen, Germany) according to the PN-EN ISO 868:2005 [49]. The hardness was indicative of an average penetration (Shore degrees on the A scale) value based on five readings from tests.

#### 2.2.3. Scanning Electron Microscopy (SEM) and Optical Light Microscopy (PLM)

The morphology of the fractured surfaces of belts samples was analyzed using a scanning electron microscope (model Mira 3, Tescan, Brno, Czech Republic) with high-resolution imaging. The fractured surfaces of belts was investigated at 23 °C, in a vacuum, using the back-scattered electron (BSE) signal, with an accelerating voltage of 15 kV. Prior to the SEM analysis, the prepared samples were coated with a thin layer (20 nm) of carbon powder. A magnification of 1000× was used. The morphology of the belts was observed using an Opta-Tech camera (Opta-Tech, Warsaw, Poland) at 20× magnification.

#### 2.2.4. Thermogravimetric Analysis

The thermogravimetric analysis (TGA) of belts was carried out using a thermogravimeter: a Netzsch TG 209 F1 apparatus (Netzsch, Ltd., Selb, Germany). The samples (about 10 ± 0.2 mg) were placed in a ceramic crucible and heated from room temperature to 800 °C at the rate of 10 °C/min in a nitrogen atmosphere. The flow gas rate was 40 mL L^−1^, and the cooling rate was set to 10 °C/min. The thermal properties of belts were evaluated by the initial decomposition temperature (*T_i_*) and the corresponding temperature at which the mass was reduced by 5% (*T*_5%_), 10% (*T*_10%_) and 50% (*T*_50%_), and temperature (*T_max_*), corresponding to the maximum mass loss rate, which was the peak of the first derivative of the rate of thermal decomposition (DTG) curve. In addition, the fraction of the solid residue at 800 °C was obtained. Furthermore, in order to obtain kinetics parameters, TGA scans at 10, 20, and 30 °C/min were carried out, and the Kissinger method [50] was applied to the obtained data, as reported elsewhere [51].

Kissinger’s method [50] uses the following Equation (1):

(1) $$ln\left(\frac{\beta }{T_{max}^{2}}\right)=-\frac{E_{a}}{RT_{max}}+ln\frac{AR}{E_{a}}$$

where *E_a_* is the apparent activation energy (kJ mol^−1^), *A* is a pre-exponential factor (s^−1^), *R* is the gas constant (8.314 J mol^−1^ K^−1^), and *T_max_* is the temperature of maximum rate of mass loss [50].

### 2.3. The Measurement Method of the Toxicity Products of Thermal Decomposition and Combustion

The principle of the method is based on the quantitative, chemical determination of the products of thermal decomposition or combustion of materials determining the toxicity of the fire environment.

Due to the lack of standards regarding the methodology for testing toxic products of thermal decomposition and the combustion of machine parts, a standard used in construction was used. About 4 ± 0.1 g of samples were prepared for testing, maintaining the uniform distribution of the represented layers. The tests were carried out in agreement with a Polish standard PN-B-02855:1988 [52] regarding the fire protection of buildings, in which the test methodology was specified. This standard coincides with a French standard NFx70-100 86 [53], German DIN 53,436 [54], and Russian GOST 12.1.044-89 [55].

**Figure 3:**
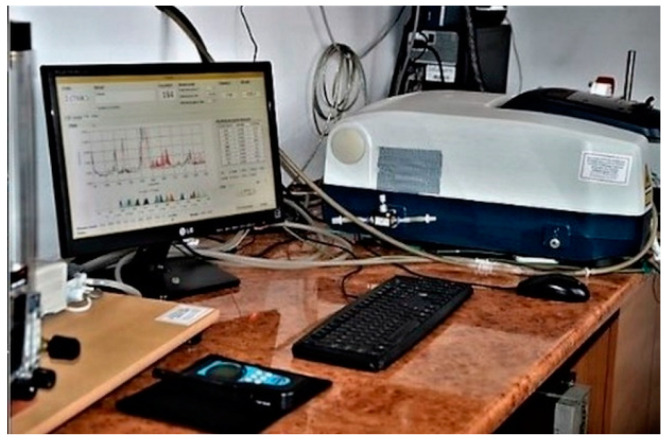
Stand for testing the toxicity of combustion products using a method according to PN-B-02855 used in Sychta Laboratory [56].

The tests were carried out using an FT-IR analyzer (FT-IR 4700 JASCO, Tokyo, Japan), which continuously measured the mass concentrations of the tested components; the test stand is shown in Figure 3.

The testing was carried out in two stages. In the first stage of testing, a qualitative analysis of the composition of products of thermal decomposition and combustion was carried out, superimposing the measured results on the standard spectra. The analysis of the tested material samples was carried out to determine carbon (C), hydrogen (H), nitrogen (N), sulfur (S), and chlorine (Cl) in order to exclude the possibility of carbon monoxide (CO), carbon dioxide (CO_2_), hydrogen cyanide (HCN), nitrogen dioxide (NO_2_), hydrogen chloride (HCl), and sulfur dioxide (SO_2_). Compounds not shown in the qualitative analysis were omitted in the quantitative test.

In the second stage of testing, the samples were subjected to quantitative analysis of emitted compounds (carbon monoxide, carbon dioxide, hydrogen cyanide, nitrogen dioxide, hydrogen chloride, sulfur dioxide) during thermal decomposition and combustion. The tests were carried out at temperatures of 450 ± 5, 550 ± 5, and 750 ± 5 °C and at an air flow rate of 100 ± 10 dm^3^/h. The air was supplied in counter-current to the movement of the furnace, moving at a speed of 20 mm/min. The time to fully move the furnace was 30 min. Samples of products of gaseous thermal decomposition and combustion for the analysis of CO and CO_2_ were taken in the succession of 7.5, 15, and 22.5 min from the beginning of the test. In case of expected emissions of hydrogen cyanide (HCN), nitrogen dioxide (NO_2_), hydrogen chloride (HCl), and sulfur dioxide (SO_2_), the products of decomposition and combustion had to be passed through the absorbing scrubbers during the slide of the furnace on the sample (30 min) and its return to the starting point (5 min). The test was performed twice at each temperature. The test was repeated a third time if the difference in results between the samples exceeded 30%.

During the tests, the concentrations of carbon monoxide, carbon dioxide, hydrogen chloride, hydrogen cyanide, nitrogen dioxide, and sulfur dioxide were determined.

The resulting concentrations of carbon monoxide, carbon dioxide, hydrogen chloride, hydrogen cyanide, nitrogen dioxide, and sulfur dioxide were noted. On this basis, the specific emissions (E) of the mentioned products of thermal decomposition and combustion were determined. Specific emission (E) means the mass of toxic products produced during thermal decomposition and the combustion of a unit mass of a material under given test conditions. Next, by associating them with the properties of concentration limits ($LC_{50}^{30}$: concentration causing the death of 50% of the population at 30 min exposure, the values of which are indicated in Table 2 for selected substances), the following toxicometric indicators are obtained:

− toxicometric indicator $\;\left(W_{LC50}\right)$ is a mass of a given material whose decomposition or combustion under test conditions produces toxic concentration limits for a given product of thermal decomposition, described by Equation (2):
  (2) $$W_{LC50}=\frac{LC_{50}^{30}}{E},\;g/m^{-3}$$
  where $LC_{50}^{30}$ is an indicator of limit concentration of products of thermal decomposition and combustion, and E is a specific emission of toxic products of thermal decomposition and combustion (g g^−1^).
− toxicometric indicator $\;(W_{LC50M})$ is a resultant of $W_{LC50}$ indicator for individual products of thermal decomposition and combustion for a given temperature determined according to Equation (3):
  (3) $$\frac{1}{W_{LC50M}}=\overset{n}{\underset{}{\sum }}{\frac{1}{W_{LC50}}}_{LC50},$$
  where n is the number of samples, and $LC_{50}^{30}$ is the concentration causing the death of 50% of the population at 30 min exposure, the values of which are indicated in Table 2.
− toxicometric indicator ($W_{LC50SM}$) is an arithmetic average of $W_{LC50M}$ indicators from individual temperatures (450, 550, and 750 °C), described by Equation (4):
  (4) $$W_{LC50SM}=\frac{1}{3}(W_{LC50M\;450{}^{\circ}C}+W_{LC50M\;550{}^{\circ}C}+W_{LC50M\;750{}^{\circ}C},$$

The *W_LC50SM_* toxicometric indicator is the basis for the classification of materials, which is presented in Table 3.

## 3. Results and Discussions

### 3.1. FT-IR Scpectoscopy Chemical Composition Analysis

**Figure 4:**
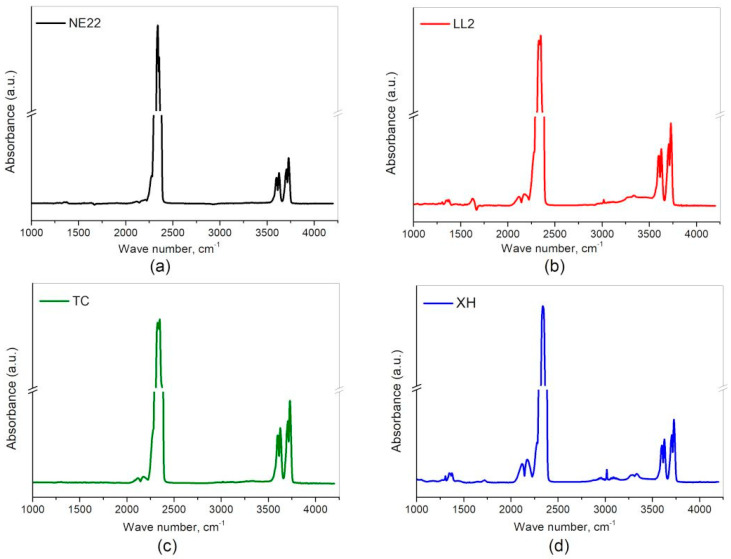
Fourier-transform infrared spectroscopy (FT-IR) spectra from the thermal decomposition and combustion of belts: (**a**) NE22, (**b**) LL2, (**c**) TC, and (**d**) XH.

After the first series of tests, Fourier-transform infrared spectroscopy (FT-IR) with was used to evaluate a qualitative identification of polymers. The obtained FT-IR spectra were interpreted with respect to the bands from the polymers in the analyzed belt. The results of FT-IR tests of the products of thermal decomposition and combustion were analyzed for NE22, LL2, TC, and XH samples. The spectra from the FT-IR analysis of combustion products of samples NE22, LL2, TC, and XH are shown in Figure 4a–d, respectively.

For the NE22 sample, the spectrum comes from the 300 s test, LL2 sample after 615 s, while for the XH after 645 s, and XH after 720 s of the test. Each IR spectrum was corrected for the presence of water and compared with a standard spectrum for the individual substances.

For the purpose of toxicity studies, the presence of SO_2_, NO_2_, NO, HCN, CO_2_, CO, HCl, HBr and HF gases was searched for on the spectra. Moreover, compared in detail the substances that were identified in the products of thermal decomposition of each belt, which illustrated in Table 4.

Figure 4a,b show peaks in the FT-IR spectrum in the wavenumber range from 1320 to 1400 cm^−1^ indicate the presence of SO_2_. A characteristic curve for the identification of NO_2_ are signals ranging from 1570 to 1650 cm^−1^ and for NO these are peaks between the wavenumber values of 1800 and 1940 cm^−1^. The presence of CO_2_ in the analyzed spectra was confirmed on the basis of the presence of the characteristic four peaks in the wavenumber range between 3560 and 3740 cm^−1^, while CO appears as two peaks between 2100 and 2200 cm^−1^. HCN is a substance whose presence is confirmed by two peaks in the range of 3200 to 3360 cm^−1^. HBr, HCl and HF appear as multiple peaks in the ranges 2400 to 2200 cm^−1^, 2650 to 3090 cm^−1^ and 3780 to 4200 cm^−1^, respectively. Their presence was not confirmed in any of the tested belt samples. Figure 4b,d show additional signals in the range of 2880 to 3150 cm^−1^, which indicate the presence of methane, however, it was not the subject of this analysis.

**Figure 5:**
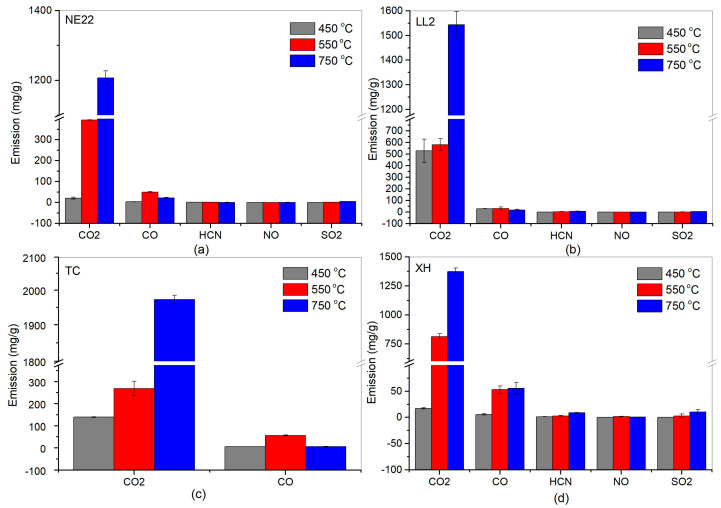
Emission of products during decomposition and combustion of belts at 450, 550, and 750 °C: (**a**) NE22, (**b**) LL2, (**c**) TC, (**d**) HX.

**Figure 6:**
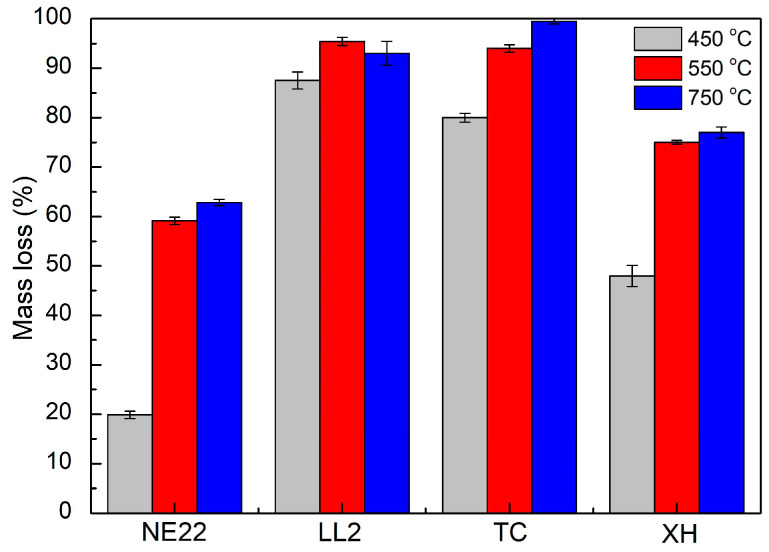
Mass loss of NE22, LL2, TC, XH belts during thermal decomposition and combustion at 450, 550, and 750 °C.

The second stage of testing concerned the qualitative analysis of products of thermal decomposition and combustion of the belts NE22, LL2, XH, and TC, for 30 min at oven temperatures of 450, 550 and 750 °C, as presented in Figure 5. Additionally, Figure 6 presents general data on thermal decomposition and combustion processes, such as the mass loss of samples.

Generally, in Figure 5, we can see that the emission of CO_2_ during the combustion of all belts increased with increasing temperature. Carbon dioxide was found to be the largest product during the first step of degradation in nitrogen for all polymers, indicating the scission of the urethane bond [57]. The CO_2_ emissions in 450 °C for the LL2 belt were 400 mg/g, which is higher than the 100 mg/g of the TC belt, indicating that the natural leather of the LL2 belt significantly increased the toxicity during combustion, which was not observed for other belts. In the case of NE22 and HX belts, the emissions of substances at the same temperature were about 0 mg/g. During the toxicity test, the mass loss of products of decomposition and combustion of belts was also analyzed.

Figure 6 shows the weight mass loss of belt samples at 450, 550, and 750 °C. The average mass loss at 450 °C for LL2 and TC belts was 92 ± 4% and 91 ± 10%, respectively. Whereas, the in case of NE22 and XH, the mass loss was 20 ± 16% and 48 ± 23%, respectively, which is lower compared to the previous belts. The tests showed that in general, the mass loss increases with the increasing measurement temperature, and the mass loss of the LL2 belt at 450 °C is higher (by approximately 77%) than that of the NE22 belt, which indicates that the LL2 belt made with multilayers leather/polyamide material decomposed faster, followed by the chloroprene/polyester material (NE22 belt), which is due to the lower initial decomposition temperature of the LL2 belt made of leather. The above results corresponded with the emissions of product data from FT-IR.

### 3.2. Values of Toxicometric Indicators Analysis

Materials used for drive or transport belts can pose a threat to human health and life due to the emission of toxic products of thermal decomposition and combustion, such as carbon monoxide, hydrogen chloride, and hydrogen cyanide. They may affect safe evacuation conditions. The emission of toxic gases by burning materials in concentrations exceeding the lethal levels for humans can effectively prevent this evacuation, causing threats to health and life.

**Figure 7:**
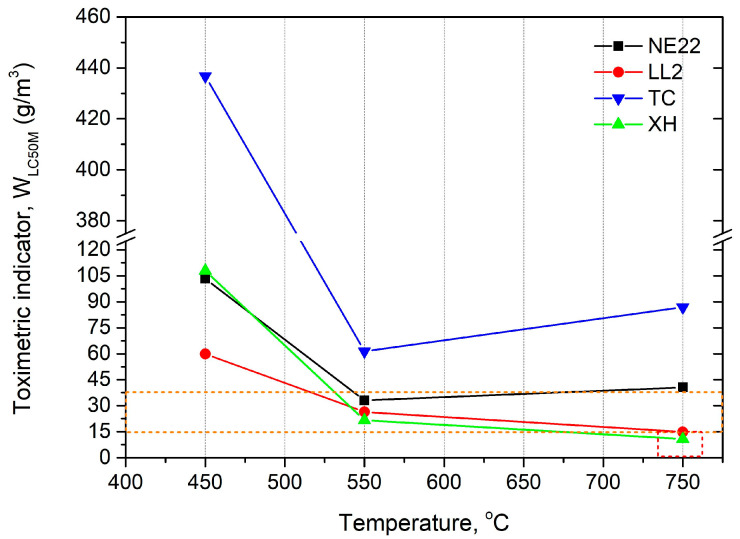
The toxicometric indicator (*W_LC50M_*) of belts as a temperature function.

The $W_{LC50M}$ toxicometric indicator being the resultant of the $W_{LC50}$ indicator of individual products of thermal decomposition and combustion for a given temperature makes it possible to determine the effect of thermal decomposition and combustion temperature on the toxic properties of the material under these conditions. The values of $\mathrm{toxicometric}\;\mathrm{indicators}\;(W_{LC50M}$) measured at 450, 550, and 750 °C for the tested belts are presented in Figure 7.

Figure 7 shows that the toxicometric indicator (*W_LC50M_*) significantly decreases with the increase of the combustion temperature and thermal decomposition tested at 450 and 550 °C. On the other hand, at 750 °C, the value of the $W_{LC50M}$ toxicometric indicators increases for NE22 and TC samples and decreases for LL2 and XH samples; however, the change is not as significant as for 450 °C.

The *W_LC50M_* results indicated that the belts made by polymer materials can affect the increase the toxicity emissions to the environment as the fire temperature increases.

### 3.3. Assessment of Products of Thermal Decomposition and Combustion

The obtained results of the toxicometric indicators (W_LC50SM_) were related to the classification criteria of toxicity of products of thermal decomposition and combustion in accordance with PN-B-02855, as presented in Table 2.

Based on the results, it can be concluded that the tested belts release substances during the thermal decomposition and combustion in concentrations, which characterizes them as moderately toxic (NE22, XH, TC) belts or toxic (LL2 belt) in the conditions of fire (Table 5).

It can be observed that belts made of classic NE22 materials (polyester/chloroprene) and most of those used today, e.g., XH and TC belts, are classified in the same group of moderately toxic materials. The results showed that the most environmentally unfavorable material among the tested belts was the LL2 belt, which is made of multilayers of leather and is characterized by classification as toxic compound material. The value of toxicometric indicator (*W_LC50SM_*) of the LL2 belt was 33.8 g/m^3^, which is lower than, e.g., NE22 and XH belts, indicating that the natural leather has a slightly reduced the W_LC50SM_ compared with the polyamide/NBR material. In the case of both NE22 and XH belts, the W_LC50SM_ was about 60 and 47 g/m^3^, respectively. This behavior is due to the chemical composition of the polymeric materials used to make the polyamide belts. It can be seen that the toxicometric indicator of the TC belt was 195 g/m^2^, which is higher than those of the other belts. Due to the TC belt being made by several polyurethane layers, it indicates the lowest toxicity during thermal decomposition and combustion.

Drive and transport belts are most often used in machines and devices in which most of the elements are made of metal and are therefore non-flammable. As a result, it can be stated that these belts during a fire can be indicated as the main emission source of toxic compounds during a fire. However, it cannot be ruled out that there are other flammable elements in the machine’s or device’s area of operation. All the machine components that are subject to burning, the equipment of a facility in which the machine is located, and the transported objects all constitute a potential fire hazard.

**Figure 8:**
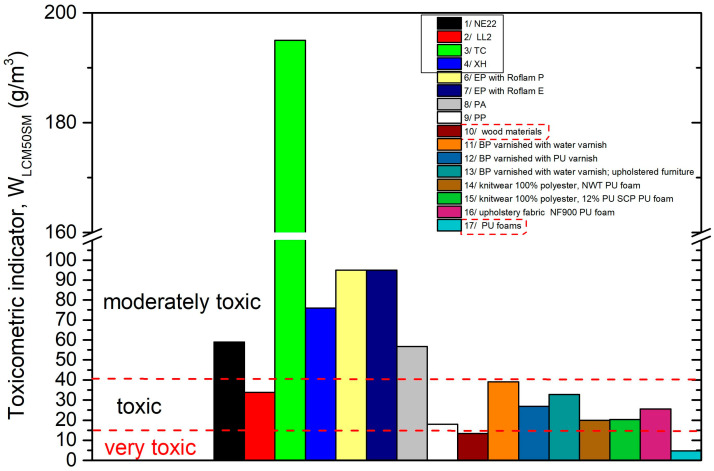
Comparison of the toxicometric indicator (W_LC50SM_) with toxicity classification of transmission and transport belts: 1—NE22, 2—LL2, 3—XH, 4—TC to selected construction materials. 5—Epidian, 6—Epidian with Roflam P, 7—Epidian with Roflam E [58], 8—polyamide (PA) [59], 9—polypropylene (PP), 10—wood-based materials [59], 11—beech plywood (BP) varnished with water varnish [59], 12—beech plywood (BP) varnished with polyurethane varnish [59], 13—birch plywood (BP) varnished with water varnish [59]; upholstery systems included in upholstered furniture, 14—knitwear 100% polyester, polyurethane foam [59], 15—knitwear 88% polyester with 12% PU SGP polyurethane foam [59], 16—upholstery fabric NF900 polyurethane foam [59]; 17—polyurethane foams [60].

The literature contains the values of toxicometric indicators (*W_LC50SM_*), which may be a component of the assessment of the cause of fires in the area of machine operation. The values of *W_LC50SM_* for drive and transport belts, epoxy resins used for finishing industrial floors [58], plastics, wood-based materials, upholstery systems included in upholstered furniture [59], and polyurethane foams [60] are presented in Figure 8.

The most dangerous are very toxic materials, which can be prohibited (when used inside a building) by legislators [61]. However, this does not mean that moderately toxic or toxic materials under fire conditions are safe. The toxicometric indicator (W_LC50SM_) averages the individual toxicometric indicators determined for all tested products of thermal decomposition and the combustion of materials at three different temperatures. Meanwhile, the quantitative assessment of toxic fire hazard should consider the amounts of individual toxic gases released in a specific stage of a fire, with particular regard to the initial phase of its development, in which effective evacuation is possible. To assess the actual toxic hazard during a fire, attention should be paid to the toxicometric indicators (W_LC50SM_). The significance of the issue is increased by the percentage of mortality caused by smoke and the toxicity of fire products, which in the case of house fires is indicated at 60%–80% [62,63,64,65], despite the existence of requirements stating that for each closed usable space (e.g., buildings and transport vehicles), it must be possible to evacuate people safely in the event of fire [66,67].

### 3.4. The Thermal Decomposition Kinetics of Belts

**Figure 9:**
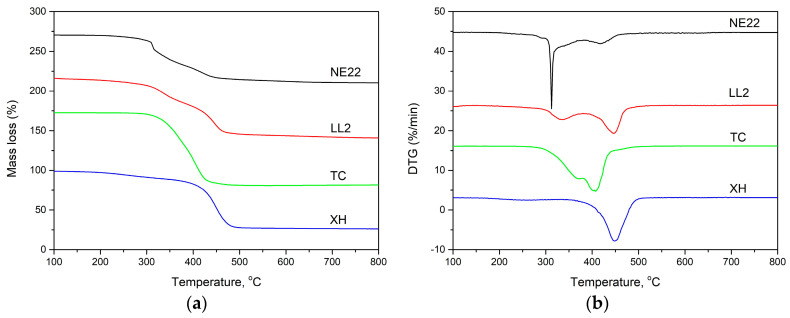
Thermogravimetric (TG) (**a**) and DTG (**b**) curves at a heating rate of 10 °C/min under nitrogen for the four kind of belts.

TGA can serve as a useful indicator of polymer decomposition and flammability behavior [18,19,40]. The thermal stability of belts was assessed by the thermogravimetric analysis in inert atmosphere, as shown in Figure 9. The characteristics TGA and DTG curves showed in Figure 9, and data are presented in Table 6. Table 6 presents the temperature 5%, 10%, and 50% mass loss at 10 °C/min (*T*_5_ wt %, *T*_10_ wt %, and *T*_50_ wt %, respectively), as well as the temperature of maximum mass loss (*T_max_*).

Thermogravimetric analysis is one of the methods used for evaluation of the decomposition of polymers at the function of temperature [19,68,69]. Figure 9 shows the TGA and TGA curves of belts. The TGA curve illustrated in Figure 9 and the data in Table 6 showed that the XH belt was decomposed in one stage in a nitrogen atmosphere. The main loss percentage happens within the temperature range 300–500 °C. In the case of LL2, TC, and NE22 samples, we can see a two-step decomposition process and its decomposition temperature starting at about 300 °C, corresponding to chloroprene degradation. The second region was the main decomposition stage, which lies in the temperature range 400–500 °C.

The values of maximum decomposition temperature (*T_max_*) at different heating rates (10, 20, 30 °C/min) are shown in Table 7.

As it can be seen from the DTG curve of TPU, the maximum rate of degradation for TPU occurred at 365 and 403 °C for the first step, *T*_1*max*_, and the second stage, *T*_2*max*_, of the degradation process, respectively. The char residue does not change much above 500 °C. Petrovic et al. directed that the degradation of TPU in the first stage occurs mainly in hard segments and depends on their content and composition [70], and the soft segments’ degradation takes part during the second step. They indicated there are three possible ways of thermal decomposition of urethane linkage: dissociation to isocyanate and alcohol, dissociation to primary amine, carbon dioxide, and olefin, as well as the formation of a secondary amine with the elimination of carbon dioxide [71]. Moreover, the thermal stability and the thermal degradation profiles of POM/TPU blends were investigated by Pielichowski et al. [22].

The initial decomposition temperatures of XH, TC, NE22, and LL2 belts for 5% mass loss are 448, 318, 286, and 115 °C, respectively, which indicated that the LL2 belt with natural leather decomposes earlier than others, e.g., the polyester/chloroprene belt (NE22 sample) and thermoplastic polyurethane (TC sample). It also means that the XH belt made with NBR/PA fabric/PA film/NBR layers was characterized by the highest thermal stability, which means reducing the flammability of the belt made of such materials [69,72].

For example, the polychloroprene of the synthetic rubber from which the NE22 belt was made is self-extinguishing compared to butadiene-based rubber (NBR). Compared to the polymer produced in the polyaddition process of a polyol with a diisocyanate, the decomposition of polyurethane begins at the temperature of 318.4 °C, i.e., 80% loss and up to 300 °C, up to 420 for all PU into monomers [66]. In the case of polymers, the significantly higher thermal stability and flammability limitations are of special importance mainly due to the risk of human behavior and emissions of harmful substances into the atmosphere of toxic products generated during thermal decomposition and burning. A very important parameter of the thermal stability of compounded polymeric materials from the point of view of their flammability is the rate of thermal decomposition (DTG_max_) [72].

The actual thermal decomposition of the NE22 belt takes place in two steps. The largest weight loss step is about 23% and occurs in the first decomposition step at 313 °C, which is mainly due to the elimination of HCl and is seen as a large peak in the DTG curve. The second weight loss step of the degradation process is about 3% and occurs at 420 °C due to the combustion of carbon. For XH and TC belts, the maximum decomposition rate is almost the same (about 10 %/min). The maximum decomposition rate of the LL2 belt is about 4 %/min, which is much lower than that of other belts, and the char residue at 800 °C of a LL2 belt is 20 mass%.

Additionally, it can be seen in Table 5 that the char residues at 800 °C with a heating rate of 10 °C/min for NE22, XH, LL2, and TC are about 40.0, 27.0, 21.0, and 9 mass %, respectively, which indicate that the NE22 belt has the best char-forming ability, and the TC polyurethane belt has the worst char-forming ability. The properties of polyurethanes (PU) strongly depend on the utilized components, including their chemical structure, type of catalyst, and the molar ratio of isocyanates to hydroxyl groups [73].

In the case of the XH belt, which is made of NBR/PA film/PA fabric/NBR, the belt is also characterized by lower values of the DTG_max_ thermal decomposition rate due to the high energy of molecular cohesion. In the first stage of decomposition of a diene rubber, the catalytic dissociation of bonds is decomposed in the macromolecule skeleton, and in the second stage, the degradation and thermal breakdown of macromolecular fragments containing butadiene units occur, which is accompanied by the release of CO, CO_2_, and HCN destruction products [74].

It can be seen that that with the increasing of the heating rate, the peak mass loss rate (*T_max_*) of the belts shifts to a higher temperature, e.g., from 448.4 to 464.5 °C for the XH belt, which shortens the time needed for the sample to reach the same temperature, and the material cannot decompose completely, leading the peak *T_max_* rate shift to a high value [19]. A similar effect was observed by Matykiewicz for the carbon-reinforced epoxy composites with the highest biochar content [75].

## 4. Conclusions

Flat drive and transport belts made of composites can become a serious toxic hazard during a fire. An improvement in the strength and durability of the belts seems to reduce this hazard; however, the results of testing showed no significant improvement in reducing the effects of a fire on the drive and transport belts. The emission of toxic products of thermal decomposition and combustion can cause a lethal hazard during a fire in places where transmission gears and conveyor belts are used. Material classification based on the toxicometric indicator (W_LC50SM_) does not provide grounds for determining the actual fire hazard. The results showed that the most environmentally unfavorable material among the tested belts was the LL2 belt, which is made of multilayers of leather and polyamide 6 characterized by classification as toxic compound material.

A useful parameter for quantifying the threat of toxic products of combustion may be the critical mass of the material, which indicates how much of the material can be used in a given room, so that in the event of a fire, the concentration limits of products of thermal decomposition and combustion are not exceeded. However, published studies indicate that drive and transport belts require further work on their chemical composition to minimize their negative impact on human health and life during a fire. Based on the TGA analysis, it was found that the XH belt has higher thermal stability in the temperature range up to 300 °C, when compared to the other TC belts made with thermoplastic polyurethane.

## Figures and Tables

**Table 1:** Physical properties of belts.

Samples	Thickness, mm	Hardness, ^o^ShA	Density, g/cm^3^
NE22	1.4 ± 0.3	82.2 ± 1.0	1.20 ± 0.04
LL2	4.0 ± 0.1	76.8 ± 2.1	1.30 ± 0.07
TC	1.4 ± 0.2	78.8 ± 0.4	1.06 ± 0.03
XH	4.0 ± 0.1	73.8 ± 0.6	1.13 ± 0.05

**Table 2:** Indicators of limit concentration of products of thermal decomposition and combustion ($LC_{50}^{30}$).

Product of Thermal Decomposition and Combustion	Value $LC_{50}^{30}$ (g·m^−3^)
Carbon monoxide (CO)	3.75
Carbon dioxide (CO_2_)	196.40
Hydrogen cyanide (HCN)	0.16
Nitrogen dioxide (NO_2_)	0.205
Hydrogen chloride (HCl)	1.00
Sulfur dioxide (SO_2_)	0.70

**Table 3:** Classification criteria for toxic products of thermal decomposition and combustion based on the value of the toxicometric indicator (*W_LC50SM_*) in accordance with PN-B-02855 [52].

WL_C50SM_	Label	Products of Thermal Decomposition and Combustion of Materials
≤15	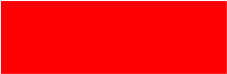	very toxic
>15, ≤40	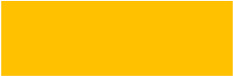	toxic
>40	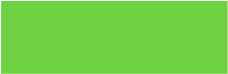	moderately toxic

**Table 4:** Comparison of identified substances in fumes after performing tests in accordance with PN-B-02855 [52].

Identified Substances	NE22	LL2	TC	XH
SO2	**+**	**+**	−	**+**
NO2	−	**+**	−	**+**
NO	**+**	**+**	−	**+**
HCN	**+**	**+**	−	**+**
CO2	**+**	**+**	**+**	**+**
CO	**+**	**+**	**+**	**+**
HCL	−	−	−	−
HBr	−	−	−	−
HF	−	−	−	−

**Table 5:** Assessment of products of thermal decomposition and combustion of belts, in accordance with the classification criterion in PN-B-02855 [52].

Type of Belt	Value of Toxicometric Indicator *W_LC50SM_* [g/m^3^]	Material Classification
NE22	59.0	moderately toxic
LL2	33.8	toxic
TC	195.0	moderately toxic
XH	46.8	moderately toxic

**Table 6:** The temperature of 5, 10 and 50 % weight loss, max. degradation rate and the fraction of the solid residue at 800 °C for belts.

Samples	*T*_5%_ [°C]	*T*_10%_ [°C]	*T*_50%_ [°C]	DTG _max rate_ (%/min)	Residue at 800 °C %
NE22	286.1	328.4	336.8	22.80/4.0	40 ± 1
LL2	115.0	264.3	428.0	3.71/7.13	21 ± 1
TC	318.4	334.7	390.8	10.92	9 ± 3
XH	448.4	459.9	464.5	10.97	27 ± 2

**Table 7:** *T_max_* for belt at different heating rates (10, 20, 30 °C/min) under nitrogen conditions.

Samples	T_1max/_*T*_2*max*_ 10 °C/min	*T*_1*max*__/_*T*_2*max*_ 20 °C/min	*T*_1*max*_/*T*_2*max*_ 30 °C/min
NE22	312.5/416.0	373.0/418.7	385.0/424.1
LL2	336.0/467.0	350.0/458.9	356.2/464.0
TC	360.0/403.4	375.0/420.8	387.0/427.1
XH	448.4	459.9	464.5
